# AKT isoforms have discrete expression in triple negative breast cancers and roles in cisplatin sensitivity

**DOI:** 10.18632/oncotarget.27746

**Published:** 2020-11-10

**Authors:** Bhumika Wadhwa, Masroor Paddar, Sameer Khan, Sameer Mir, Philip A.Clarke, Anna M. Grabowska, Devanahalli G. Vijay, Fayaz Malik

**Affiliations:** ^1^ Academy of Scientific and Innovative Research (AcSIR), Ghaziabad 201002, India; ^2^ Cancer Pharmacology Division, CSIR-Indian Institute of Integrative Medicine, Srinagar 190005, India; ^3^ Cancer Biology, Division of Cancer and Stem Cells, School of Medicine, Queen’s Medical Centre, University of Nottingham, Nottingham NG7 2RD, UK; ^4^ HCG Cancer Centre, Ahmedabad 380060, India

**Keywords:** AKT isoform, CSCs, ABCG2, drug resistance, TNBCs

## Abstract

AKT, a serine threonine kinase, exists in three different isoforms and is known for regulating several biological processes including tumorigenesis. In this study, we investigated the expression and net effect of the individual isoforms in triple negative breast cancers and response to cisplatin treatment using cellular, mice models and clinical samples. Interestingly, analysis of the expressions of AKT isoforms in clinical samples showed relatively higher expression of AKT1 in primary tissues; whereas lung and liver metastatic samples showed elevated expression of AKT2. Similarly, triple-negative breast cancer cell lines, BT-549 and MDA-MB-231, with high proliferative and invasive properties, displayed higher expression levels of AKT1/2. By modulating AKT isoform expression in MCF-10A and BT-549 cell lines, we found that presence of AKT2 was associated with invasiveness, stemness and sensitivity to drug treatment. It was observed that the silencing of AKT2 suppressed the cancer stem cell populations (CD44^high^ CD24^low^, ALDH1), mammosphere formation, invasive and migratory potential in MCF-10A and BT-549 cells. It was further demonstrated that loss of function of AKT1 isoform is associated with reduced sensitivity towards cisplatin treatment in triple-negative breast cancers cellular and syngeneic mice models. The decrease in cisplatin treatment response in shAKT1 cells was allied with the upregulation in the expression of transporter protein ABCG2, whereas silencing of ABCG2 restored cisplatin sensitivity in these cells through AKT/SNAIL/ABCG2 axis. In conclusion, our study demonstrated the varied expression of AKT isoforms in triple-negative breast cancers and also confirmed differential role of isoforms in stemness, invasiveness and response towards the cisplatin treatment.

## INTRODUCTION

Breast cancer is the second-most lethal cancer in women around the world [1]. Despite recent advances in tumor therapy, the main concern remains metastasis and relapse. Metastasis is a complex, multi-functional, and tightly regulated process in which cancer cells lose their apical-basal polarity and extensive adhesions of the basement membrane integrity and spread from the primary tumor site to invade the surrounding tissue, thereby forming secondary tumors [2–4].

Targeting dysregulated tumor driving pathways in human cancer has been a promising tool in cancer therapy and one such is the PI3K/AKT/mTOR signalling pathway. Protein kinase-B (PKB)/ AKT, a serine/threonine kinase has emerged to play a central role in regulating pleiotropic cellular functions such as cell growth and proliferation, cell survival, energy metabolism, and resistance to anticancer therapeutics. Receptor tyrosine kinases are phosphorylated in response to ligand stimulation, which then activates phosphatidylinositol 3-kinase (PI3K) to drive subsequent phosphorylation by PDK1 on a threonine residue in the catalytic domain (T308 on AKT1, T309 on AKT2 and T305 on AKT3), but also requires phosphorylation on a serine residue which is located at the hydrophobic C-terminal region (S473 on AKT1, S474 on AKT2 and S472 on AKT3) by the mammalian target of rapamycin complex 2 (mTORC2); other protein kinases have also been found capable of phosphorylating the same Ser residues thereby activating AKT and other downstream signalling loop [5, 6]. It is well known that all the three AKT isoforms are highly homologous in their amino acid sequences (~80%) and display similar substrate specificity namely an N-terminal pleckstrin homology (PH) regulatory domain, a catalytic domain in the middle, and a C-terminal region necessary for the induction and maintenance of kinase activity but are encoded by separate genes [7, 8].

Despite the mounting body of evidence for isoform-specific regulation, it is still unclear how the three isoforms of AKT transmit stimulus to unique downstream targets to attain distinct outcomes. In fact, components within the PI3K/AKT/mTOR loop are frequently dysregulated in human breast cancers [9, 10]. For example, triple- negative breast cancers (TNBC) harbor mutations in the tumor suppressor genes, PTEN and p53 [11]. PTEN has been shown to be one of the most commonly altered genes, resulting in gain-of-function in AKT isoforms. Notably, these alterations arise either from the net increase in intrinsic signals or can be provoked in an extrinsic manner by the tumor microenvironment. Based on *in vitro* and *in vivo* studies, it is becoming uncertain which of the three AKT isoforms is indeed relevant in driving neoplastic phenotypes.

Amongst the known neoplastic characteristics, AKT kinase is involved in EMT, DNA damage repair, cell death inhibition which endows increased aggressiveness and resistance of drugs [12–14]. A study by Gagnon et al. (2004) explored cisplatin resistance via AKT2 and AKT3 isoforms that lead to malignant human uterine cancer cells [15]. It has become evident that AKT drives epithelial–mesenchymal transition (EMT) and is linked with increased tumor invasion, growth and poor prognosis [16]. Nevertheless, to understand the significance of the outcomes driven by the AKT isoforms, with respect to normal versus malignant breast cancer, it is important to characterize which AKT isoform leads to oncogenesis or exerts self-contradictory effects, both promoting and impeding neoplastic phenotypes.

Therefore, we sought to determine the isoform-specific functions of AKT in triple-negative breast cancers. To this end, we modulated AKT isoform expression in a human mammary nonmalignant immortalized cell line, MCF-10A, and malignant breast cancer cell line, BT-549 by knocking down endogenous AKT isoforms using short hairpin RNA (shRNA). Our *in vitro* and mice xenograft experiments demonstrated that AKT isoforms variedly influence the cellular proliferation, invasiveness, stemness and response against cisplatin treatment. Interestingly, analysis of triple-negative breast cancer clinical samples from primary and metastatic site have shown differential expression of AKT isoforms. These studies highlight the role of specific AKT isoforms in invasiveness and poor response to cisplatin treatment in Triple-negative breast cancers that needs to be evaluated further for the development of isoform specific inhibitors for better clinical outcome.

## RESULTS

### Elevated expression of AKT1/2 isoforms in triple-negative breast cancer subtype

**Figure 1:**
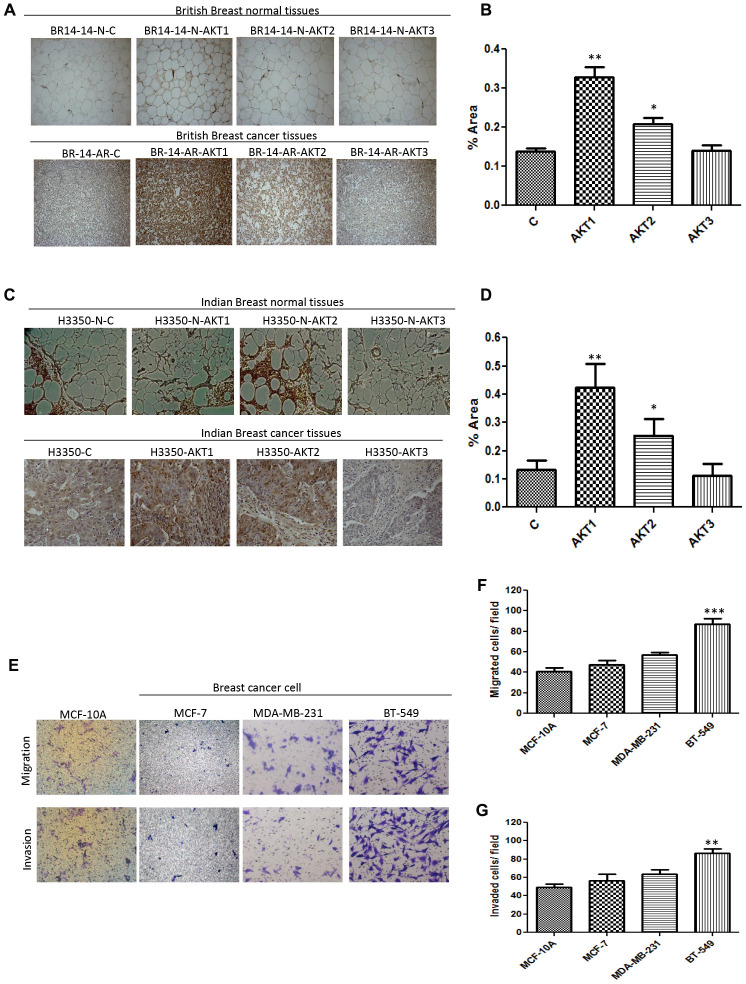
Correlation of AKT isoform expression with malignant breast cancer. (**A**) British triple-negative breast cancer with normal counterpart tissues were immunostained using AKT1, AKT2 and AKT3 antibodies. High expression levels of AKT1 and AKT2 were shown in 5 of 5 triple-negative breast cancer tissues. Representative images of one case in breast normal tissues (upper) and breast cancer tissues (lower) are shown. (**B**) Quantification of 9 cases (5-British and 4-Indian). (**C**) Tissue block from Indian triple negative breast cancer (upper) with its normal counterpart (lower) showed high expression of AKT1 and AKT2 in 4 of 4 triple-negative breast cancer cases. Representative images of one case are shown. (**D**) Quantification of 4 British PDX cases. Photographs were taken by DM500 microscope (Leica, New York, USA) equipped with DP71 digital imaging system (Leica). (**E**–**G**) Representative images of migration and invasion in various breast cancer cell lines (magnification, X100), and data are presented as the mean ± standard deviation of three independent experiments.

Hyperactivation of AKT kinase remains one of the driving signals of Triple-negative breast cancers. Our earlier studies [17] and those from other groups [18, 19] have shown activation of AKT signalling and mesenchymal features in triple-negative cellular and animal models. Keeping in view the non-redundant role of AKT isoforms, we evaluated the isoform-specific expression in triple-negative breast cancer tissue samples (FPPE) of the patients from different ethnicity and geographical locations. We performed immunohistochemistry on British and Indian origin tissue samples containing primary human triple-negative breast cancer with corresponding normal tissues. Our results from 5/5 of primary triple-negative samples of British origin, 4/4 of British patient derived xenografts (PDX) and 4/4 of Indian origin showed elevated expression of AKT1 and AKT2 isoforms, while the expression of AKT3 was not significant (Figure 1A–1D). We further evaluated the expression of AKT isoforms in different breast cancer cell lines and found that AKT1 and AKT2 were strongly expressed in the cells of Triple-negative cancer subtype, BT-549 and MDA-MB-231, compared to their corresponding expression in luminal MCF-7 and non-tumorigenic MCF-10A cells (Supplementary Figure 1A–1C). Before further exploration of the role of AKT isoforms in aggressive properties of Triple-negative subtype of breast cancers, our comparative analysis showed that BT-549 bears strong invasive and migratory potential compared to MCF-7 and MCF-10A (Figure 1E–1G).

### AKT1 promotes, while AKTs 2 and 3 attenuate, cell proliferation

Several studies have shown that AKT1 leads to mammary tumor growth [20–22]. We generated stable clones of BT-549 and MCF-10A cells using overexpression of AKT1, AKT2 and AKT3 isoforms that were confirmed by analyzing mRNA expression (Supplementary Figure 2A and 2B). Similarly, stable clones of BT-549 and MCF-10A cells using knockdown of AKT1, AKT2 and AKT3 isoforms were analyzed for mRNA and protein expression (Supplementary Figure 2C and 2D). Time-dependent (24, 48, 72 and 96 h) observation indicated that knockdown using shAKT1 (shAKT1) drastically attenuated cell proliferation in MCF-10A and BT-549 cells (Supplementary Figure 2E and 2F). The Effect on cell proliferation was further confirmed by The evaluating the expression of cell proliferation marker, Proliferating cell nuclear antigen (PCNA) by immunoblotting. In MCF-10A and BT-549 cells, PCNA analysis in knockdown clones of AKT isoforms revealed its reduced expression in shAKT1 cells, while expression was elevated in shAKT3 cells. To further confirm the role of a single isoform in the increase or decrease in cell proliferation, we performed dual silencing (shAKT1/2, shAKT2/3 and shAKT3/1); both in MCF-10A and BT-549 cells. It was observed that double knockdown of AKT2 and AKT3 (shAKT2/3) leads to increased cell proliferation and expression of PCNA (Supplementary Figure 2G and 2H). These results demonstrate that the presence of AKT1 drives cell proliferation, while the role of AKT2 and AKT3 remained anti-proliferative.

### AKT2 over-expression is allied with invasive and metastatic triple-negative breast tumors as observed in 2D and 3D models

**Figure 2:**
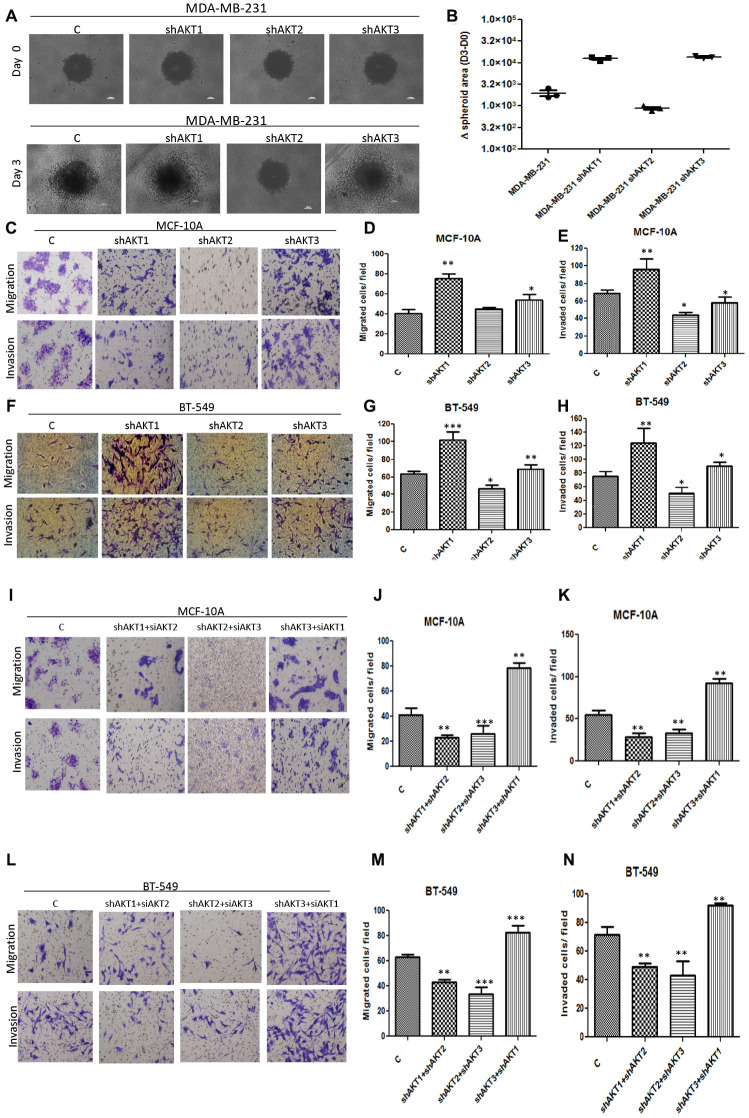
Correlation of AKT2 with cell aggressiveness. (**A**–**B**) 3D spheroids of MDA-MB-231 (top panel) at day 0 and (bottom panel) at day 3 during invasion. (**C**–**H**) Effect of invasion and migration in MCF-10A and BT-549 knockdown AKT isoforms. (**I**–**N**) Representative images of invasion and migration analysis in dual AKT isoform silencing. The results are shown as mean ± SD of one representative experiment (from three independent experiments) performed in triplicate. Statistically significant differences (^***^) *p* < 0.001, (^**^) *p* < 0.01, (^*^) *p* < 0.05 are indicated.

**Figure 3:**
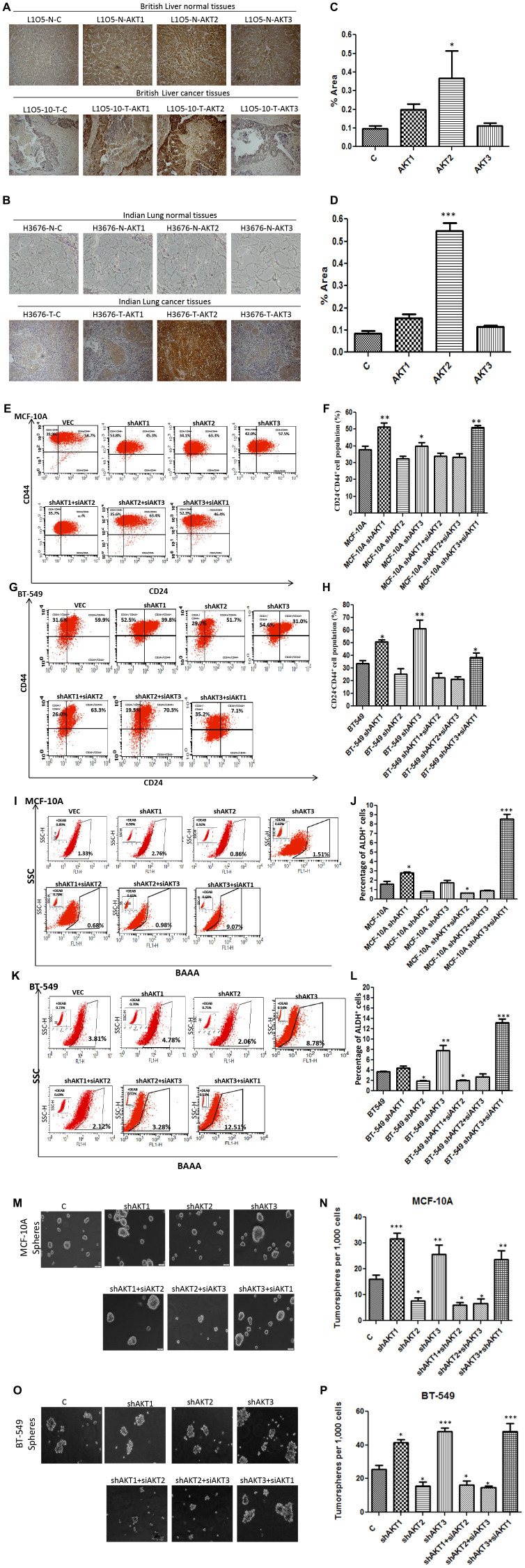
AKT2 promotes cancer stem cells and contributes to metastasis. (**A**) Immunohistochemistry of liver tissue arrays (secondary site) against AKT1, AKT2 and AKT3 antibodies. High expression levels of AKT2 were seen in both British (cancer and normal) and (**B** and **D**) Indian (cancer and normal) lung tissues. (**C**) Quantification of 3 British metastatic samples. (**D**) Quantification of 1 Indian metastatic case. Photomicrographs were taken by DM500 microscope (Leica, New York, USA) equipped with DP71 digital imaging system (Leica). (**E**–**F**) FACS analysis and quantitation of CD44^high^CD24^low^ cell population in MCF-10A and (**G**–**H**) BT549. (**I**–**J**) FACS analysis and quantitation of ALDH^+^ percentage in MCF-10A and (**K**–**L**) BT-549. All the results show the mean of three independent experiments. Error bars indicate SEM. ^*^ *p* < 0.05, ^**^ *p* < 0.01, ^***^ *p* < 0.001. (**M**–**N**) Representative images of the mammospheres formed by knockdown variants of AKT isoforms in MCF-10A. The comparison is made between 6 different variants of MCF-10A with that of MCF-10A cells. (**O**–**P**) Representative images of the mammospheres formed by knockdown variants of AKT isoforms in BT-549. The comparison is made between six different variants of BT-549 with that of BT-549 cells.

Since Triple-negative breast cancers are highly invasive and metastatic, we evaluated the 3D invasive properties of individual knockdown of AKT isoforms in MDA-MB-231 cells and found that on day 3, cell clones expressing AKT2 isoform (shAKT1 or shAKT3) showed significant invasive characteristics, whereas shAKT2 isoform did not undergo any change (Figure 2A and 2B). In addition, we evaluated the invasive and migratory properties of AKT isoforms in BT-549 and MCF-10A cells in 2D cell culture. In both MCF-10A and BT-549, knockdown of AKT2 reduced, while that of shAKT1 enhanced, the invasive and migratory properties of these cells (Figure 2C–2H). On the contrary, overexpressed AKT2 was found to exhibit enhanced invasive and migratory properties in both MCF-10A and BT-549 cells (Supplementary Figure 3A–3F). To further, unravel whether these properties require the presence of two isoforms or only an individual isoform, we performed dual isoform silencing/overexpression in both BT-549 and MCF-10A cells. Dual silencing of isoforms (shAKT1/2, shAKT2/3, shAKT1/3) showed that the solo expression of AKT2 isoform in shAKT1/3 confers invasive and migratory properties on cells (Figure 2I–2N). On the contrary, dual overexpression of isoforms (AKT1/2, AKT2/3, AKT1/3) revealed that the presence of AKT2 expression in AKT1/2 and AKT2/3 refers to increased invasion and migration in these cells (Supplementary Figure 3G–3L). To further, understand the functional role of each isoform on the metastatic phenotype, we investigated expression of AKT isoforms in the clinical specimen metastasized to the liver and lung. Both the British and Indian tumor histology patterns revealed higher expression of AKT2 while expressions of AKT1 and AKT3 were not significant when observed in both liver and lung metastasized FPPE samples (Figure 3A–3D). These results were in accordance to that of IHC staining of clinical samples, showing that among the three isoforms, AKT2 has a possibly major role in metastasis.

A large body of evidence has now shown that in tumors only a fraction of cancer cells known as cancer stem cells (CSCs) have the ability to undergo epithelial-to-mesenchymal transition (EMT), a pre-requisite for becoming invasive and migratory, hence forming micro-metastases [23, 24]. We performed flow cytometric analysis (FACS) of stem cell markers (CD44/CD24, ALDH) and observed that silencing of AKT2 isoform reduced, while AKT3 knockdown increased, the CD44^+^/CD24^-^ CSCs population both in MCF-10A and BT-549 cells. To further confirm the role of individual isoforms, a double knockdown revealed that AKT2 is associated with increased CD44^+^/CD24^-^ CSCs as evidenced in shAKT1/3 dual silenced MCF-10A and BT-549 cells (Figure 3e-h). Another stem cell-specific assay ALDEFLUOR (ALDH^+^), revealed that both MCF-10A and BT-549 cells with silenced AKT2 isoform have a reduced population of ALDH^+^ CSCs. Dual silencing of AKT isoforms revealed that AKT2 in shAKT1/3 cells results in a 9-fold increase (9.07%) in ALDH^+^ cells in MCF-10A compared to its base value 1.33% (Figure 3i and j). Similarly, in BT-549 cells, double knockdown of AKT1/3 lead to 3 fold (12.5%) increase of ALDH^+^ CSCs compared to control cells with 3.8% population (Figure 3K and 3L). These results further substantiated that AKT2 leads to the expansion of ALDH^+^ CSCs, while silencing of AKT2 reduces the CSC population.

Another important property of metastasis initiating cells is the self renewal and survival under matrix detachment conditions. To investigate the role of AKT isoforms in self renewal under non-adherent conditions, cells were grown in ultralow adhesion plates. Following re-culturing of spheroids in stem cell medium after 10 days, they remained in sphere shape after several generations. In both MCF-10A and BT-549 cells, knockdown of AKT2 reduced mammospheres formation whereas overexpressed AKT2 had an effect quite contrary to the knockdown variants (Figure 3M–3P and Supplementary Figure 3M–3P). Similarly, dual isoform silencing/overexpression in both MCF-10Aand BT-549 cells, demonstrated that the solo expression of AKT2 isoform in shAKT1/3 and presence of AKT2 isoform expression in dual overexpression of AKT1/2 and AKT2/3 confers to enhanced mammospheres formation (Figure 3M-3P and Supplementary Figure 3M–3P). Collectively, our results have shown the involvement of AKT2, but not AKT1 and AKT3, in cancer cell migration and invasion thereby acquiring the characteristics of cancer stem cell.

### Silencing of AKT1 isoform is correlated with the G2/M arrest

**Figure 4:**
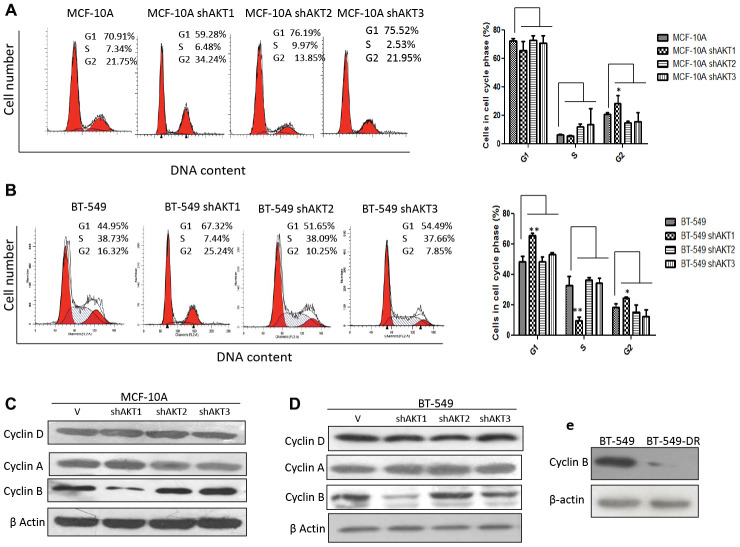
Knockdown of AKT1 resulted in prolonged G2 arrest. (**A**–**B**) Cell cycle analysis of AKT isoforms by knockdown of parental cell line (A) MCF-10A and (B) BT-549. (**C**–**D**) Western blot images of cell cycle proteins in knockdown variants of AKT isoforms in (C) MCF-10A, (D) BT-549, (**E**) Western blot image of cyclin B expression in BT-549 and BT-549-DR cells. All the results show the means of three independent experiments. Error bars indicate SEM. Data were analyzed using Student’s t test. ^*^ *p* < 0.05, ^**^ *p* < 0.01.

We next further evaluated the relationship between cell cycle and stem-like cells using FACS and found that the stem-like cell population within knockdown of AKT1 (shAKT1) both in MCF-10A and BT-549 cells causes G2 arrest as demonstrated by cell cycle analysis (Figure 4A and 4B). This was further confirmed since protein levels of cyclin B, a marker which is highly expressed in M phase was significantly lower in MCF-10A shAKT1, BT-549 shAKT1 and BT-549 DR (drug resistant) as compared with wild-type controls while no change in the expression of cyclin D (G1 phase) or cyclin A (S phase) was seen (Figure 4C–4E). The downregulation of cyclin B supported the idea that AKT1 knockdown cells have a prolonged G2 phase. Furthermore, an increase in the phosphorylation site of Cdk1 at Tyr15 (Y15) was observed in AKT1 knockdown in both MCF-10A, BT-549 and drug (cisplatin) resistant BT-549 cells (Supplementary Figure 4A–4C). It is well known that the phosphorylation of Cdk1 at Y15 by members of the Wee/Mik1/Myt1 protein kinase family renders Cdk1 in an inactive state, thereby resulting in G2 arrest [25]. Also, Cdk1 is regulated by Cdc25C [26]. We next analyzed the protein expression level of Cdc25C in different subcellular localizations and found that Cdc25C was higher in the cytoplasmic fraction than in nuclear fraction of shAKT1 MCF-10A and BT-549 whereas shAKT2 and shAKT3 showed the opposite cellular distribution (Supplementary Figure 4D and 4E). Similar results were observed in cisplatin resistant BT-549 cells (Supplementary Figure 4F). This indicates that the retention of Cdc25C in the cytoplasm of shAKT1 cells leads to inactive Cdk1 and the prolonged G2 arrest while retaining Cdc25C in the nucleus of both MCF-10A and BT-549 shAKT2 and shAKT3 cells showed that AKT1 regulates the G2/M transition.

### Inhibition of AKT1 induces expression of efflux pump ABCG2 leading to cisplatin resistance

**Figure 5:**
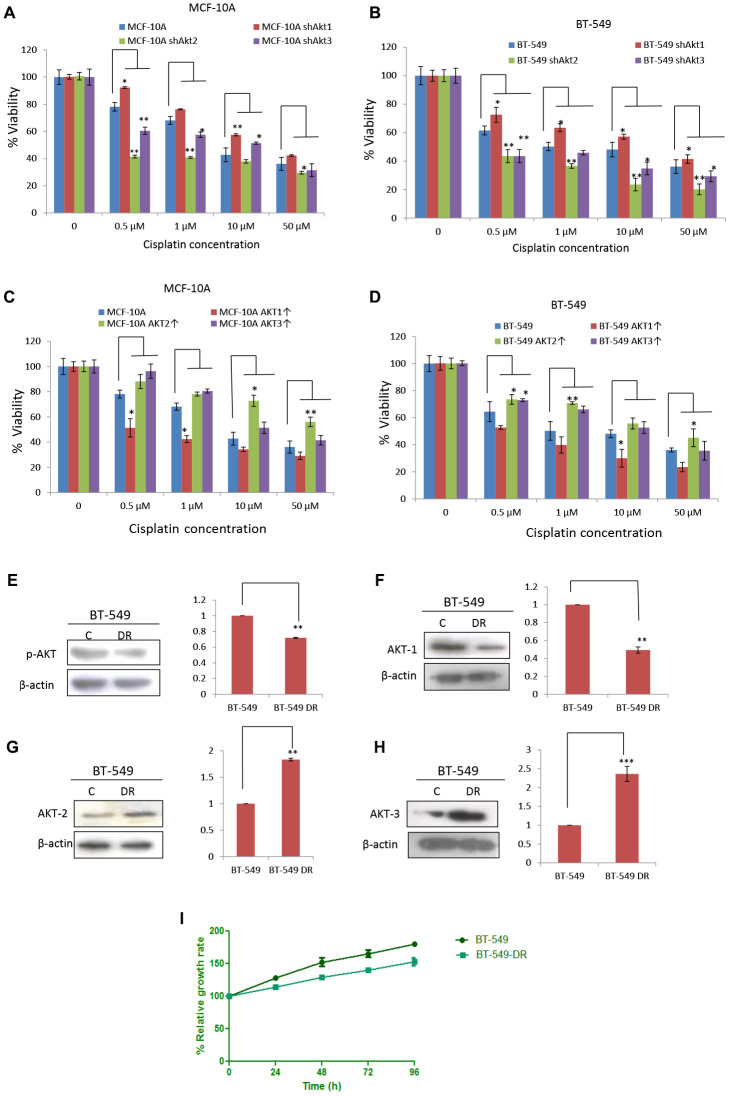
Knockdown of AKT1 abrogated sensitivity towards cisplatin. (**A**) Survival rates of MCF-10A shAKT1, MCF-10A shAKT2, MCF-10A shAKT3 and their control cells treated with different concentrations of cisplatin for 48 h. The comparison is made between three different clones of MCF-10A with that of MCF-10A. (**B**) Survival rates of BT-549 shAKT1, BT-549 shAKT2, BT-549 shAKT3 and their control cells treated with different concentrations of cisplatin for 48 h. The comparison is made between three different clones of BT-549 with that of BT-549 cells. (**C**) Survival rates of MCF-10A overexpressed AKT isoforms variants and their control cells treated with different concentrations of cisplatin for 48 h. The comparison is made between three different clones of MCF-10A with that of MCF-10A cells. (**D**) Survival rates of BT-549 overexpressed AKT isoforms variants and their control cells treated with different concentrations of cisplatin for 48 h. The comparison is made between three different clones of BT-549 with that of BT-549 cells. (**E**–**H**) Western blotting images and quantitation of (E) p-AKT, (F) AKT1, (G) AKT2, (H) AKT3 in BT-549 and BT-549-DR cells. β-actin was used as a loading control and bands seem to have no background as very less exposure time was given during X-ray film development. (**I**) Survival rates of BT-549 and BT-549-DR at different time intervals.

The survival rates of knockdown and overexpression of all the three AKT isoforms, both in MCF-10A and BT-549, were measured after 48 h cisplatin treatment. At all concentrations of cisplatin tested (0, 0.5, 1, 10, and 50 μM) cells with AKT1 knockdown showed reduced sensitivity to cisplatin treatment, whereas AKT2 knockdown cells were most sensitive to cisplatin treatment (Figure 5A and 5B). On the contrary, AKT1 overexpressed cells were found to be most sensitive to cisplatin treatment at all concentrations of cisplatin tested (0, 0.5, 1, 10, and 50 μM) in both MCF-10A and BT-549 (Figure 5C and 5D). Decreased expression of p-AKT and AKT1 in BT-549 DR cells was also associated with decreased survival (Figure 5E–5I). After having observed decreased chemosensitivity and diminished apoptosis caused by AKT1 knockdown, we examined the expression levels of procaspase-3 and PARP-1 in different cisplatin-treated AKT isoforms knockdown in both MCF-10A and BT-549. As shown in (Supplementary Figure 5A and 5B), no procaspase-3 and PARP-1 cleavage are seen in AKT1 knockdown (shAKT1) cells when compared with that of the control, shAKT2 and shAKT3 cells.

**Figure 6:**
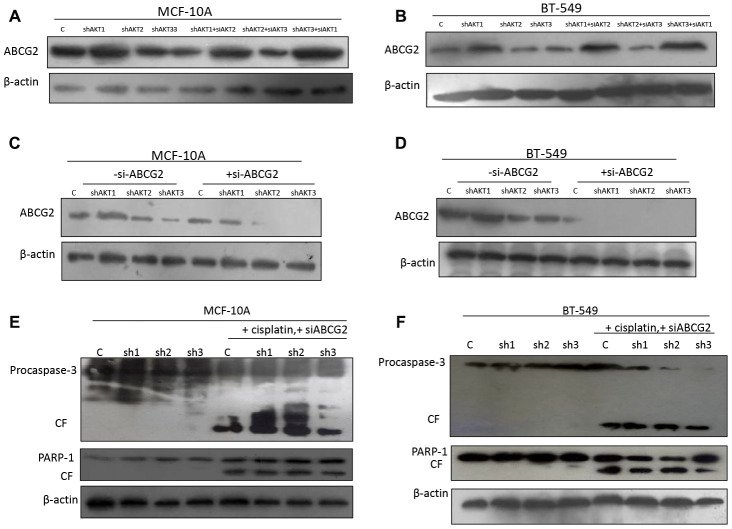
AKT1 effect on ABCG2 while abrogating cisplatin sensitivity. (**A**–**B**) Western blotting images of expression of P-gp inhibitor (BCRP/ABCG2) in an individual/dual isoform silencing in (A) MCF-10A and (B) BT-549 cells. (**C**–**D**) ABCG2 specific siRNA were used to silence the expression of ABCG2 in (C) MCF-10A and (D) BT-549 cells. (**E**–**F**) Both MCF-10A and BT-549 knockdown of AKT isoforms were further treated with cisplatin for 48 h. Protein lysates were analyzed by western blotting for the indicated proteins and the chemiluminiscent signals were captured using x-ray film. Some areas in the picture seemed lighter than the background, may be due to uneven exposure to x-ray film during development. All the results show the means of three independent experiments. Error bars indicate SEM. Data were analyzed using Student’s *t* test.

We hypothesized that one of the factors contributing to decreased sensitivity to cisplatin in AKT1 knockdown cells is ATP-binding cassette sub-family G member 2 (ABCG2), which pumps cisplatin out of the cell. Our results demonstrated that the drug efflux protein, ABCG2 is upregulated following dual silencing of AKT1/3 (shAKT1/3) in MCF-10A and BT-549 (Figure 6A and 6B). To further confirm the results, we knocked down expression of ABCG2 in individual knockdown of AKT isoforms (shAKT1, shAKT2, and shAKT3) both in MCF-10A and BT-549 cells through siRNA and subjected these cells to cisplatin treatment. We next examined that ABCG2 silencing promoted the apoptotic effect of cisplatin even following knockdown of AKT1 (shAKT1) as evidenced by increased procaspase-3 and PARP-1 cleavage (Figure 6C–6F). To generalize our findings with other p-gp pump drug efflux inhibitors, we similarly inhibited the expression of ABCG2 with zosuquidar and similar expression levels of apoptotic proteins were seen when treated simultaneously with cisplatin (Supplementary Figure 6A–6D). Altogether, this indicates that AKT2 plays an important role in the regulation of drug efflux pump.

### AKT1-knockdown induces EMT via AKT, SNAIL and ABCG2 signaling axis in breast cancer cells

**Figure 7:**
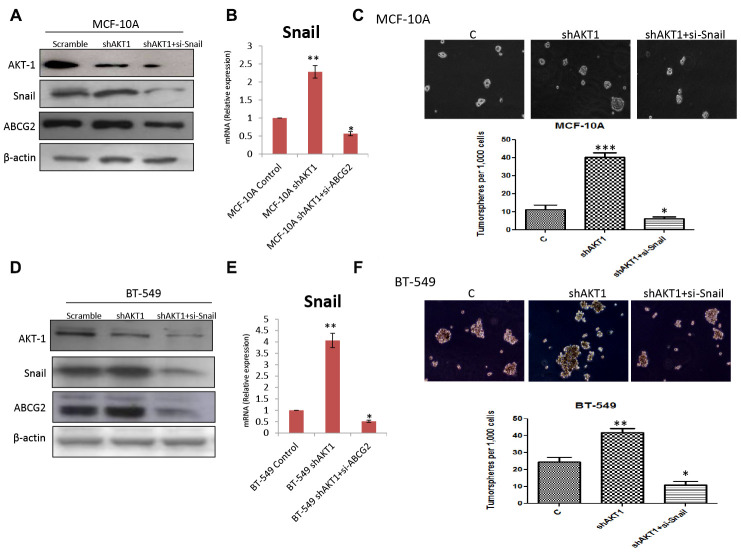
Knockdown of Snail in shAKT1 (**A**) MCF-10A and (**D**) BT-549 cells decreased the expression of ABCG2. qPCR analysis confirming the enhanced expression of Snail in (**B**) MCF-10A shAKT1 and (**E**) BT-549 shAKT1 cells. Knockdown of Snail in shAKT1 (**C**) MCF-10A and (**F**) BT-549 declined the number of sphere formation ability. Data are reported as mean ± SEM, with significance indicated by ^*^ *p* < 0.05, ^**^ *p* < 0.01 and ^***^ *p* < 0.001.

Since knockdown of AKT1 in both MCF-10A and BT-549 cells lead to the activation of transcription factor SNAIL, ABC transporter ABCG2, and acquisition of resistance towards cisplatin, we next examined whether down-regulation of SNAIL in AKT1-knockdown cells was associated with an expansion of the cancer stem cell population. As shown in Figure 7A–7D, treatment with siRNA targeting an EMT regulator SNAIL in MCF-10A shAKT1 and BT-549 shAKT1, decrease in the ABC transporter ABCG2 with an inhibition of clone-forming ability at the single cell level was seen. Importantly, treatment with siRNA targeting SNAIL in these cells in combination with cisplatin treatment induced caspase-3 and PARP-1 cleavage thereby making MCF-10A shAKT1 and BT-549 shAKT1 sensitive to cisplatin (Supplementary Figure 7A and 7B). This further suggests that by knockdown of AKT1 (shAKT1) cells acquire EMT and cisplatin resistance through AKT/SNAIL/ABCG2 axis.

### Targeting AKT2 suppresses tumor growth in a triple-negative breast cancer syngeneic model

**Figure 8:**
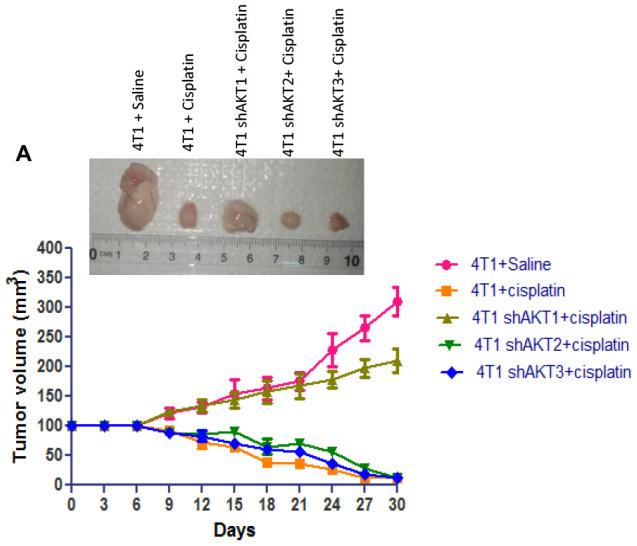
Down-regulation of AKT2 decreases tumorigenic capacity. (**A**) Tumorigenic capacity *in vivo*. 1 × 10^6^ cells were injected into the right flank of female Balb/c mice. There seem some contrasting regions of very bright and dull on the same photograph. This was because excised tumors were placed on the laminated surgical pads and pictures were taken by smartphone camera inside the biosafety cabinet with lights on. Representative photo shows differential volume of tumor at 30 days after cisplatin treatment and tumor burden was assessed after every 3 days in injected animals, for a total of 30 days. *Error bars* represent mean ± S. D. versus control.

To explore the dominant AKT isoform for treatment of drug resistant triple negative breast cancer; we transduced the mouse triple negative breast cancer cell line, 4T1, with shRNA targeting AKT1/2/3 and subcutaneously implanted 1 × 10^6^ cells into female Balb/c mice for investigating *in vivo* efficacy. 4T1 cell line establishes highly proliferative and metastatic tumors in immunocompetent BALB/c mice and also models human triple-negative breast cancer [27]. Co-treatment with cisplatin in mice carrying xenografts formed from knockdown of AKT2 (4T1 shAKT2) and AKT3 (4T1 shAKT3) in 4T1 cells dramatically attenuated tumor formation in mice after two weeks as compared with that of mice with knockdown of AKT1 (4T1 shAKT1) treated with cisplatin (Figure 8A). These results imply that targeting AKT2 isoform will prove to be the effective treatment for cisplatin-resistant triple negative breast cancer as AKT2 isoform is required in maintaining the breast cancer stem cell population.

## DISCUSSION

Among patients with different breast cancer subtypes, metastatic triple-negative breast cancer have the worst prognosis with median survival of only 13 months [28]. However, constitutive active gain of function mutation in AKT kinase is presumed to be the driver of tumorigenesis in triple negative breast cancers [29]. In the present study, we have demonstrated that how AKT isoforms play a distinct role in cell proliferation, invasion, stemness and response to drug (cisplatin) treatment. We used triple-negative breast cancer cell lines, mouse xenografts, and clinical samples from two different ethnic backgrounds of UK and India. Our data showed that specific expression of AKT2 isoform is associated with invasiveness and stem cell characteristics whereas AKT1 expression is responsible for the proliferative potential of cells. Notably, human triple-negative breast FFPE samples isolated from primary tumor site of the patients of two different ethnic backgrounds showed relatively elevated expression of AKT1 isolated from primary tumor site of patients, whereas higher expression of AKT2 was observed in lung/liver metastatic samples. It remains to be determined what was the influence of tumor microenvironment on the activation of AKT isoform, at metastatic niches. Recent studies in triple-negative breast cancers have highlighted a link between EMT and CSC formation and postulated that CSCs often exhibit EMT properties including tumor metastasis, therapeutic resistance and disease recurrence [30]. Our findings reveal that the AKT1 isoform plays a role in promoting cell proliferation as was further demonstrated that AKT1 silencing led to the G2/M arrest in both MCF-10A and BT-549 cells. On the other hand, it was observed that AKT2 isoform is allied with the acquisition of stem/ progenitor properties associated with tumor invasion and migration both in 2D and 3D cellular models. Our experiments demonstrated that the expression of AKT2 isoform led to the expansion of CSCs population as evidenced by CD44/24, ALDH^+^ analysis and formation of tumorospheres. The role of AKT2 in invasive and CSC expansion properties was further confirmed by individual and dual silencing of the other two isoforms, AKT1 and AKT3. Several studies have shown that expression of the Snail family (Snail and Slug) transcription factor has a key role in EMT, stemness and drug resistance. To identify a direct correlation between proliferation and stemness, we subjected MCF-10A and BT-549 individual AKT isoform knockdown cells to cisplatin treatment and found that the silencing of AKT1 isoform confers poor sensitivity towards cisplatin insult by conferring protection against apoptosis as observed by PARP cleavage and Caspase-3 expression. On the other hand, it was interesting to observe that expression of AKT1 was downregulated, while that of AKT2 and AKT3 were elevated in cisplatin resistant cells thereby stating that both AKT2 and AKT3 isoforms may redundantly compensate for each other in response to drug (cisplatin) treatment. Besides overexpression of AKT isoforms also reveal the possibility of functional redundancy between AKT2 and AKT3 specifically to cisplatin treatment. Our results demonstrated that expression of drug efflux pump ABC transporter (ABCG2) was found to be enhanced in shAKT1 cells, while double knockdown of isoforms showed presence of AKT1 isoform negatively regulates the expression of ABCG2, thus rendering cells sensitive to cisplatin mediated apoptosis. This was further confirmed by silencing ABCG2 through siRNA and small molecule inhibitor zosuquidar thus rendering AKT1 silenced cells responsive to cisplatin-induced apoptosis. Several studies have shown that AKT mediated CCL21 induced upregulation of Snail confers stem cell properties and chemo-resistance in colorectal cancers [31], and multidrug resistance in MCF-7 cells [32]. Our experiments have demonstrated that the silencing of AKT1 increased the transcription and translational expression of Snail and tumorosphere formation. Furthermore, knockdown of Snail using siRNA caused reduced expression of ABCG2 and a diminished number of tumorospheres, thereby indicating that AKT1 is negatively regulating the stemness and cisplatin resistance in triple-negative breast cancers. Since, use of cisplatin is one of the main therapeutic strategies to treat the triple-negative breast cancer patients [28, 33], our studies have demonstrated that loss of function of AKT1 isoform in particular has ability to affect cancer cell sensitivity against the treatment. In this direction our syngeneic mice xenograft data using 4T1 cells with silenced AKT isoforms, further confirmed that loss of AKT1 diminishes the therapeutic response against cisplatin treatment, thus indicating that expressions of AKT2/3 confer poor response against cisplatin treatment. This indicates that loss of AKT1 or dysfunctional AKT1 expression can impede the treatment efficacy of cisplatin. In conclusion, the present work elucidated expressions of AKT isoforms vary in primary and secondary sites of TNBCs, which needs to be further validated by taking large sample size. These studies unraveled the specific roles of AKT isoforms in stemness, invasions and therapeutic response of cisplatin in TNBCs, therefore suggesting that it is imperative to precisely design isoform-specific inhibitors in the treatment of aggressive triple-negative breast cancers.

## MATERIALS AND METHODS

### Reagents and chemicals

RPMI-1640, DMEM, 3-(4, 5, - dimethylthiazole-2-yl)-2, 5 diphenyltetrazolium bromide (MTT), phenylmethanesulfonyl fluoride (PMSF), cis-diammineplatinum (II) dichloride, EGF, cholera toxin, hydrocortisone, insulin were purchased from Sigma-(Aldrich, MS, USA. DMEM/F12, antibiotic-antimycotic (100×), horse serum, Lipofectamine 2000 was obtained from GIBCO Invitrogen Corporation USA. Antibodies of Akt1, Akt2, Akt3, Vimentin were purchased from Cell signaling technology, Massachusetts, USA. Antibodies for caspase-3, PARP-1 and β -Actin were from Santa Cruz Biotechnology, Texas, USA. Electrophoresis reagents, reagents for protein estimation were from Bio-Rad Laboratories, California, USA. Polyvinyldifluoride (PVDF) membrane was purchased from Millipore, Massachusetts, USA. Aldefluor assay buffer (#01701) and DEAB (#01705) were from STEM-CELL Technologies (Vancouver, BC, Canada). Zosuquidar (LY335979) was obtained through Kanisa Pharmaceuticals Inc (Irvine, CA, USA). Transfection reagent, medium and ABCG2 siRNA (sc41151) were purchased from Santa Cruz Biotechnology.

### Cell culture, growth conditions, and treatment

MCF-10A, the spontaneously immortalized human normal epithelial cell line, was acquired from and authenticated by American Type Culture Collection (Manassas, VA, USA) and the cells were grown in DMEM/F12 medium (GIBCO) supplemented with 5% horse serum, EGF (20 hg/ml), insulin (10 mg/ml), hydrocortisone (500hg/ml), and cholera toxin (100 hg/ml). Human breast cancer cell line BT-549, were maintained in RPMI1640 supplemented with 10% FBS, 100U penicillin G, and 100 μg streptomycin per ml. Human kidney cell line Phoenix™ Ampho were purchased from ATCC and were grown in DMEM with an additional 2mM L-glutamine. Cells were grown in CO_2_ incubator (Thermocon Electron Corporation, TX, USA) at 37 °C with 95% humidity. Cisplatin [*cis*-diammineplatinum (II) dichloride] was obtained from Sigma-Aldrich and dissolved in 0.15 M NaCl.

### Subcellular fractionation

The preparations of cytosolic and nuclear fractions were the same as previously described [34].

### Western blot analysis

The preparation of cell lysates and western blot analysis were the same as previously described [35].

### Quantitative real-time PCR analysis (qRT-PCR)

Total RNA was extracted with RNeasy mini kit (Qiagen) and 1.0 mg of which served as the templates for synthesizing complementary DNA (cDNA) by using SuperScript III reverse transcriptase (Invitrogen). The resultant cDNA together with the Taqman commercially available gene expression assays for *Akt1* (Hs00178289), *Akt2* (Hs01086102), *Akt3* (Hs00987350), *Twist1* (Hs01675818) were used for real-time PCR analysis on a 7500 fast real-time PCR machine (Applied Biosystems). PCR was performed at 95 °C for 15 s and 60 °C for 60 s for 40 cycles. Actin beta (ACTB) was used as an internal standard to normalize mRNA levels for differences in sample concentration and loading. Quantitative PCR reactions were performed in triplicate.

### Transfection and cell infection

pBabe-Puro, shAkt1, shAkt2, and shAkt3 were a kind gift from Ricardo Gargini, Universidad Autónoma de Madrid. To obtain infectious retrovirions prior to transduction into target cells, retroviral vectors were first introduced into packaging cells known as Phoenix™ Ampho by Lipofectamine 2000 transfection method. 24 hours later, the medium was replenished and the resultant supernatant was collected twice at 12-hour intervals and each harvest was immediately overlaid on the target cells which were seeded in a six-well plate. Afterwards, the infected cells were selected with 2.5 mg/ml puromycin for 7~10 days.

### Gene silencing with siRNA

ABCG2-specific siRNA was transfected into MCF-10A and BT-549 by using lipofectamine reagent from Santa Cruz Biotechnology according to the manufacturer’s instructions. Briefly, 1 × 10^6^ cells were seeded in 3ml of transfection medium containing ABCG2 siRNA or control siRNA (80 nM) for 8 h, followed by replacement of transfection medium with the complete respective medium containing 2× fetal bovine serum for BT-549, 2× horse serum for MCF-10A and 2× antibiotic solution. Cells were then harvested for experimental purposes during 24-72 h.

### Induction of cisplatin resistance in BT-549 cells

To generate cisplatin resistant BT-549 cell line, parental cell line BT-549 was given continuous exposure to cisplatin (Sigma-Aldrich) following IC_50_ values which were obtained from initial dose-response studies of cisplatin (0.1 μM–100 μM) over 72 h. Initially, BT-549 was treated with cisplatin (IC_50_) for 72 h and the medium was replaced with fresh media to allow cells to recover for further 72 h. This process of development was carried out for approximately 6 months, followed by re-assessing of IC_50_ concentrations. Cells were then maintained at the new cisplatin IC_50_ concentrations for further 6 months.

### Transwell motility and invasion assays

Cells obtained from sub-confluent culture were dissociated by trypsinization and resuspended in starvation medium. We used 24-well transwell chambers (8 mm pore size; BD Biosciences). 1× 10^5^ of the resultant cells per well were seeded in 100μL of serum-free medium in the upper chamber whereas the lower chambers were filled with 600μL of complete medium as a chemoattractant at the same time. After 24 h at 37 °C incubation, the non-motile cells remaining at the upper surface of the membrane were swapped off with cotton swabs while the motile cells on the lower surface of the membrane were fixed with 4% paraformaldehyde and stained with 0.1% crystal violet solution. The number of migrated cells were photographed by inverted fluorescence microscope. The transwell invasion assay was identical as described above, except that inserts were coated with 100 μL Matrigel (BD Bioscience) diluted to 1 mg/mL for 6 h at 37 °C incubation prior to the seeding of the cells onto the membrane, followed for 24 h.

### 3D tumor spheroid invasion assay

1 × 10^4^ cells per well were seeded in 200μL into ultra-low attachment (ULA) 96-well round bottom plates and kept for 4 days (in order to obtain 300-500 μm diameter) at 37 °C incubation. After 3 days, place the ULA 96-well plate on ice and gently remove 100 μl/well of growth medium from the spheroid plates without disturbing the spheroids. Using ice-cold tips, add 100 μl of thawed BMM (bone marrow-derived macrophages) into each well. Using a sterile-needle, remove the bubbles, if present. Check the position of spheroids using a microscope. If the spheroids are not in a central position, centrifuge the plate at 300 × g for 3 min at 4 °C. Transfer the plate to an incubator and allow BMM to solidify. After 1 h, gently add 100 μl/well of complete growth medium. The plate was further incubated for 48 h and cells were photographed by inverted fluorescence microscope.

### Cell cycle analysis

The preparation of samples for cell cycle analysis were the same as previously described [35].

### Flow cytometry analysis

Cells at the logarithmic growth phase were washed once with phosphate-buffered saline (PBS) and then digested with 0.25% trypsin. Detached cells were washed with PBS for three times containing 1% FCS and 1% penicillin/streptomycin (wash buffer), followed by suspension in 100 ul of wash buffer, and then stained with anti-CD44-PE and anti-CD24-FITC or stained with their respective isotype controls were added to the cell suspension at concentrations recommended by the manufacturer and incubated at room temperature for 40 min. The samples were then washed thrice with the wash buffer and finally resuspended in 200 μl of wash buffer, and analyzed on FACS Calibur (BD, San Jose, CA, USA). The expression ratio of (CD44/CD24) in different subtypes were calculated from the percentage of CD44 and CD24 positive subpopulations in the flow cytometry analysis.

### ALDEFLUOR assay

The ALDEFLUOR kit (Stem Cell Technologies, Durham, NC, USA) was used to analyze the population with high ALDH enzymatic activity according to the manufacturer’s instructions. Briefly, the cells were incubated in the ALDEFLUOR assay buffer containing ALDH substrate (BAAA, 1 mmol/l per 1 × 10^6^ cells), and incubated for 40 min at 37 °C. In each experiment, a corresponding sample of cells was stained under identical conditions, using 50 mmol/l of diethylaminobenzaldehyde (DEAB), a specific ALDH inhibitor, as a negative control.

### Mammosphere/Tumorsphere assay

Single cells were plated on ultra-low attachment plates at a density of 1 × 10^5^/ml and grown for 7 days in a mammocult medium (Stem Cell Technologies). Then the primary spheres were dissociated into single cell suspension and plated at a density of 5 × 10^3^–1 × 10^4^/ml for the subsequent passages. Secondary spheres were counted after 7 to 10 days in culture.

### Survival assay

Approximately 7,000 cells at their exponential growth stage were seeded in each well of 96-well plates in triplicates. On the next consecutive day, cells were treated with various concentrations of cisplatin (0, 0.5, 1, 10 and 50 μM) for 48 h, and then viable cells were quantified using MTT.

### Immunohistochemistry

British triple negative breast cancer tissue arrays (*n* = 5), normal tissues (*n* = 5), British Patient derived xenografts (PDX) (*n* = 4) and breast metastasized to liver with the corresponding normal tissues (*n* = 3) were obtained with National Research Ethics Service (NRES) approval (NRES REC 10/H0405/6) and informed patient consent in an anonymized manner at the Nottingham University Hospitals NHS Trust and Indian triple negative breast cancer tissue arrays (*n* = 4) with its corresponding normal tissues (*n* = 4), breast metastasized to lung with its normal tissue (*n* = 1) were obtained with Healthcare Global Cancer Center ethical approval (AOA 31423/1217), Ahmedabad with patient consent. Immunohistochemistry were performed as previously described [36].

### Tumor Studies

Female Balb/c mice (18-23 g) were procured from the animal facility of the institute (Institutional Animal Ethical Committee approval (IAEC no.1361/73/8/18) and our ethical protocols follow the ARRIVE Guidelines. Mice were distributed into 5 different groups (Group I- Control, Group II- 4T1, Group III- 4T1 shAKT1, Group IV- 4T1 shAKT2 and Group V- 4T1 shAKT3) with 5 mice in each group. Knockdown of 4T1- AKT1/2/3 cells (1 × 10^6^) were suspended in PBS, mixed with an equal volume of Matrigel, and subcutaneously inoculated into the right flank of each animal. Once tumor volumes reached almost 100 mm^3^, group I was administered a single intravenous of 0.2 ml normal saline as a control and all the other groups received a single intravenous dosage of cisplatin (10 mg/Kg). Tumors volumes were calculated as described earlier [37] by vernier calliper every 3 days. On day 30, all animals were sacrificed and tumors were excised.

### Statistical analysis

Data are presented as means of three similar experiments and the error bars represent the standard deviation (SD) between the experiments. Comparisons among data sets were made by using Bonferroni method and p value < 0.05 was considered to be significant with ^***^ *p* < 0.001, ^**^ *p* < 0.01, ^*^ *p* < 0.05.

## SUPPLEMENTARY MATERIALS



## Abbreviations

CSC: Cancer stem cells
DMEM: Dulbecco’s Modified Eagle’s Medium
EGF: Epidermal growth factor
EMT: Epithelial-mesenchymal transition
FACS: Fluorescence-activated cell sorting
FFPE: Formalin-fixed paraffin-embedded
TNBC: Triple-negative breast cancer
RPMI: Roswell Park Memorial Institute Medium
RT-qPCR: Quantitative reverse-transcription polymerase chain reaction
siRNA: Small interfering RNA
