# Supplementary material for: AKT isoforms have discrete expression in triple negative breast cancers and roles in cisplatin sensitivity
Source: Oncotarget. 2020 Nov 10;11(45):4178–94. doi: 10.18632/oncotarget.27746 (PMC7665233; doi:10.18632/oncotarget.27746)
Supplement: Supplementary file 1 [file oncotarget-11-4178-s001.pdf]

AKT isoforms have discrete expression in Triple Negative breast cancers and roles in cisplatin sensitivity

SUPPLEMENTARY MATERIALS

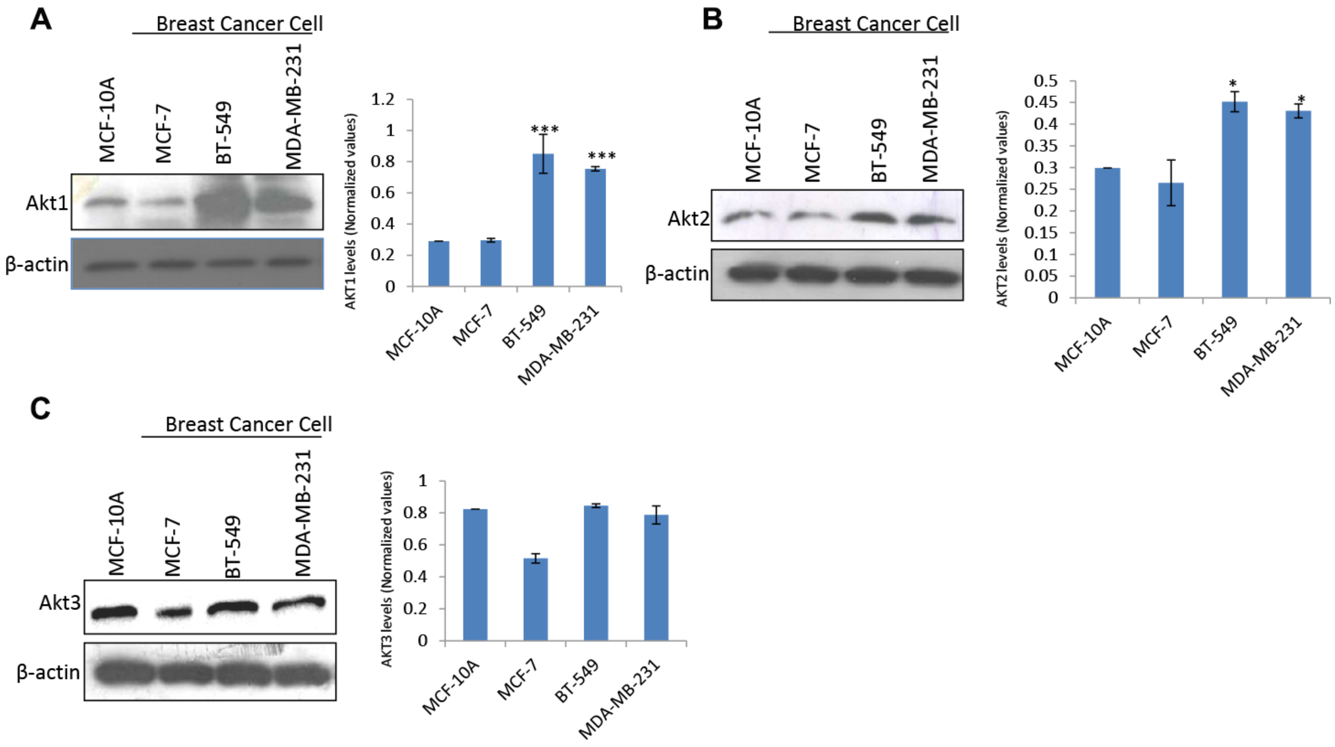

Supplementary Figure 1: Western blot images correlating the expression of AKT1, AKT2 and AKT3 isoforms with malignant breast cancer. (A-C) AKT isoform differential expression between breast normal and cancer cell lines. Data are presented as the mean ± standard deviation of three independent experiments.

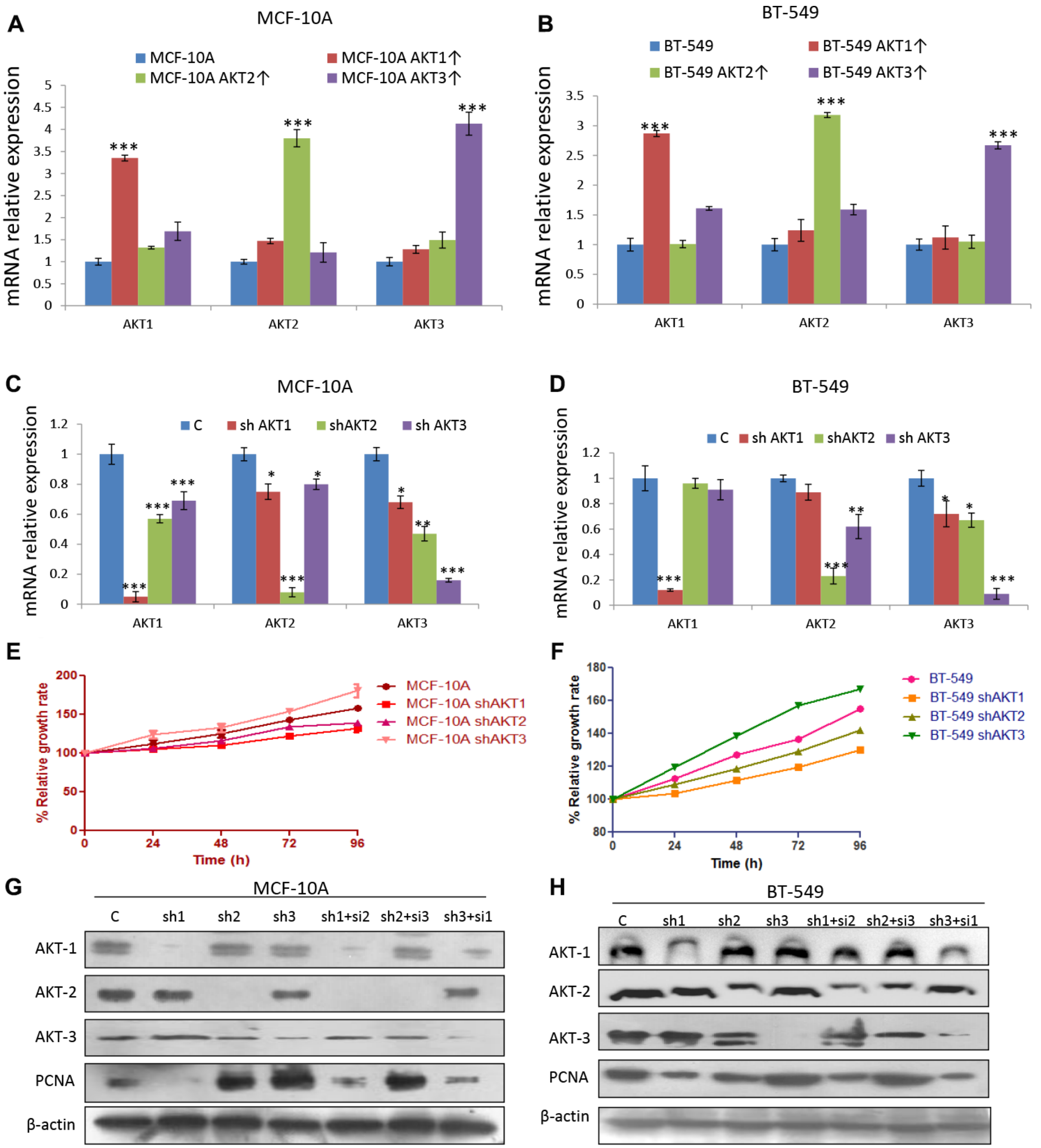

Supplementary Figure 2: AKT1 promotes cell proliferation in MCF-10A and BT-549 cell lines. qPCR analysis confirming the overexpression of AKT1, AKT2 and AKT3 isoforms expression in (**A**) MCF-10A and (**B**) BT-549 cells. qPCR analysis confirming the shRNA-mediated downregulation of AKT1, AKT2 and AKT3 expression in (**C**) MCF-10A and (**D**) BT-549 cells. (**E**) Cell viability was analyzed by MTT at different time intervals in MCF-10A and in (**F**) BT-549. (**G**) Western blot analysis of PCNA in individual and dual AKT isoform silencing in MCF-10A and (**H**) BT-549. All the results show the mean of the three independent experiments. Error bars indicate SD. Columns, mean; bars, SD with $^{***}p < 0.001$, $^{**}p < 0.01$, $^{*}p < 0.05$ versus control.

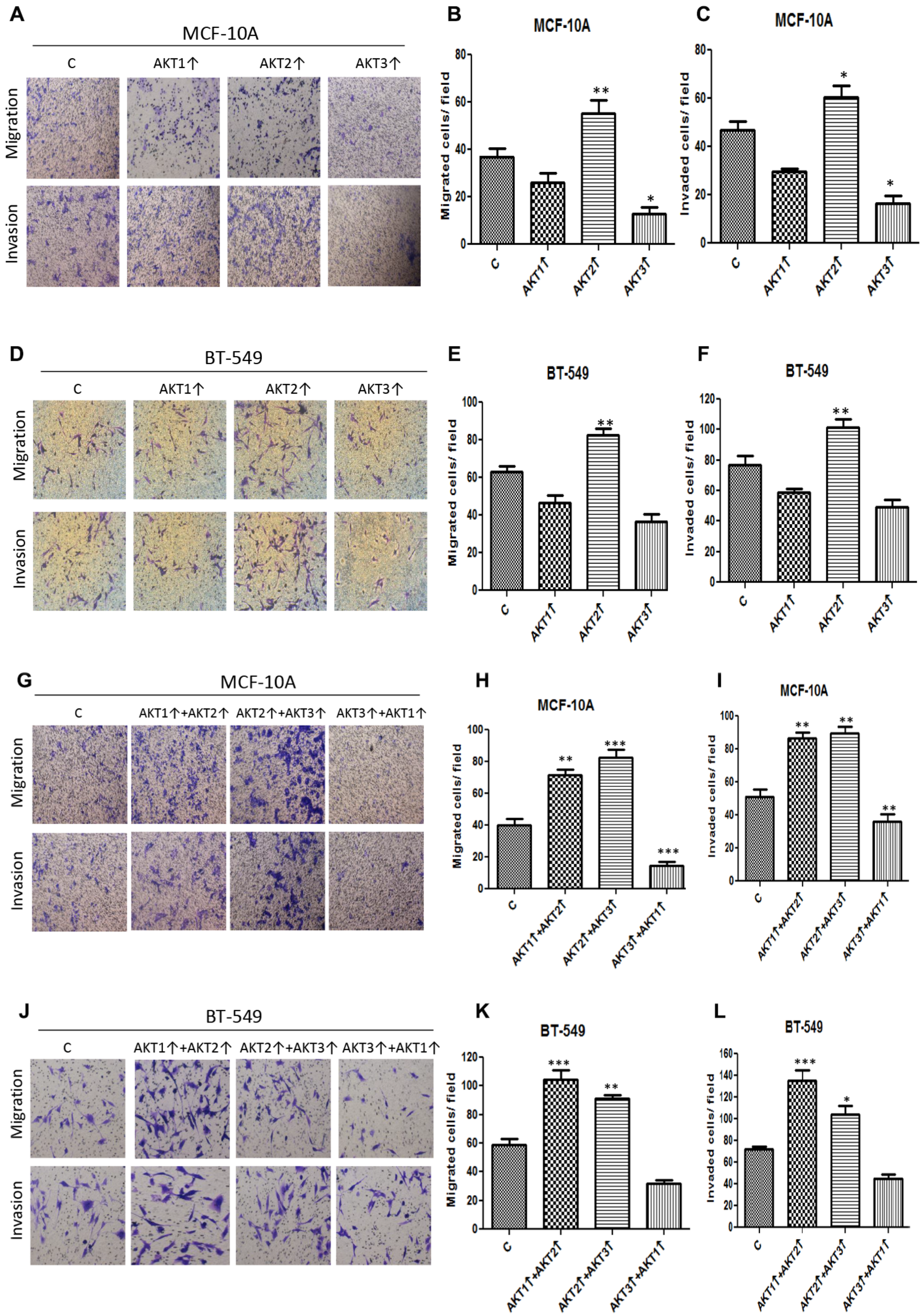
A
MCF-10A
C
AKT1↑
AKT2↑
AKT3↑
Migration
Invasion
B
MCF-10A
Migrated cells/ field
**
*
C
MCF-10A
Invaded cells/ field
*
*
D
BT-549
C
AKT1↑
AKT2↑
AKT3↑
Migration
Invasion
E
BT-549
Migrated cells/ field
**
F
BT-549
Invaded cells/ field
**
G
MCF-10A
C
AKT1↑+AKT2↑
AKT2↑+AKT3↑
AKT3↑+AKT1↑
Migration
Invasion
H
MCF-10A
Migrated cells/ field
**
***
***
I
MCF-10A
Invaded cells/ field
**
**
**
J
BT-549
C
AKT1↑+AKT2↑
AKT2↑+AKT3↑
AKT3↑+AKT1↑
Migration
Invasion
K
BT-549
Migrated cells/ field
***
**
L
BT-549
Invaded cells/ field
***
*

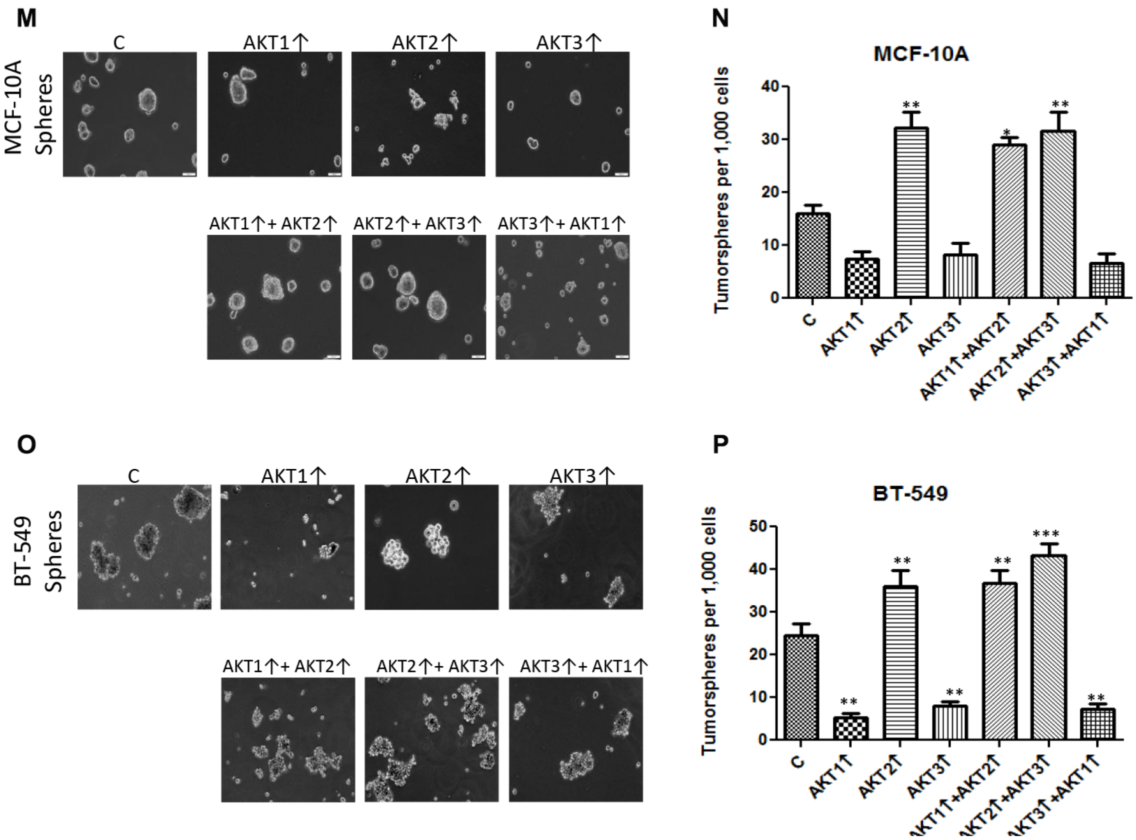

Supplementary Figure 3: AKT2 isoform is correlated with aggressiveness of cells and contributes to metastasis. (**A**–**F**) Effect of migration and invasion in MCF-10A and BT-549 cells with the overexpressed AKT isoforms. The comparison is made between three overexpressed variants with that of control cells. (**G**–**L**) Representative images of migration and invasion analysis in dual AKT isoform overexpression in MCF-10A and BT-549 cells. The comparison is made between three dual overexpressed variants with that of control cells. (**M**, **N**) Representative images of the mammospheres formed by overexpressed variants of AKT isoforms in MCF-10A. The comparison is made between six different variants of MCF-10A with that of MCF-10A cells. (**O**, **P**) Representative images of the mammospheres formed by overexpressed variants of AKT isoforms in BT-549. The comparison is made between six different variants of BT-549 with that of BT-549 cells. The results are shown as mean ± SD of one representative experiment (from three independent experiments) performed in triplicate. Statistically significant differences (***) $p < 0.001$, (**) $p < 0.01$, (*) $p < 0.05$ are indicated.

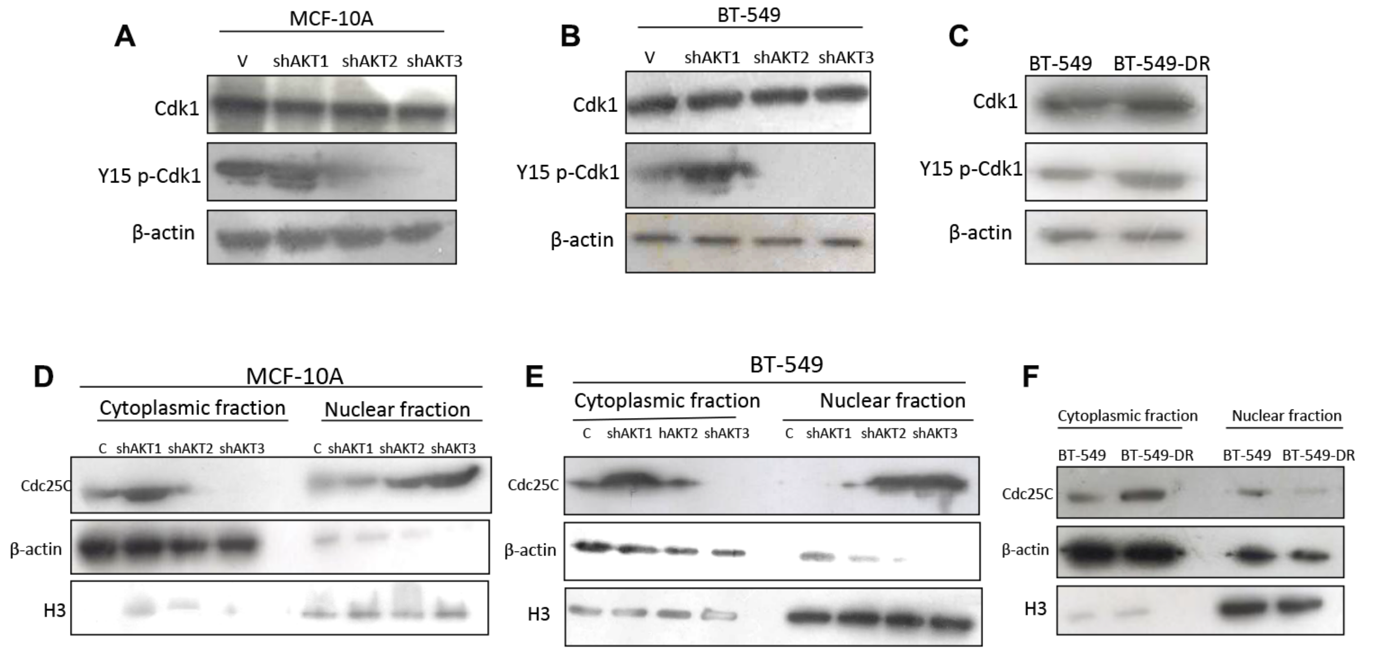

Supplementary Figure 4: AKT1 knockdown leads to prolonged G2 arrest. (**A**–**C**) Western blotting images of Cdk1 and Y15p-Cdk1 in (A) MCF-10A AKT isoforms knockdown, (B) BT-549 AKT isoforms knockdown, (C) BT-549 and BT-549-DR cells. (**D**–**F**) Western blot analysis of Cdc25C in subcellular fractions of (D) AKT isoforms knockdown in MCF-10A, (E) AKT isoforms knockdown in BT-549, (F) BT-549 and BT-549-DR cells.

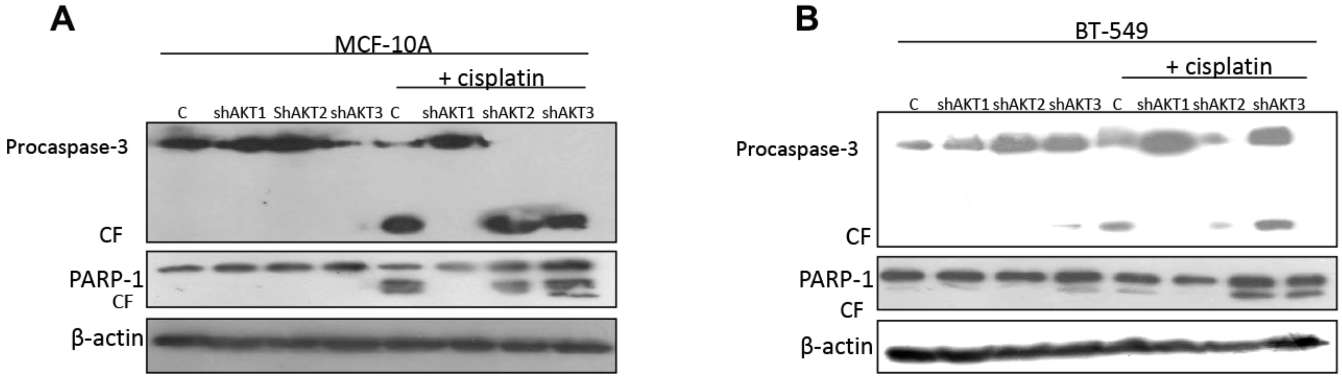

Supplementary Figure 5: AKT1 knockdown abrogated cisplatin sensitivity. (**A**, **B**) Western blot analysis of caspase-3 and PARP-1 in presence and absence of cisplatin in (A) MCF-10A and (B) BT-549.

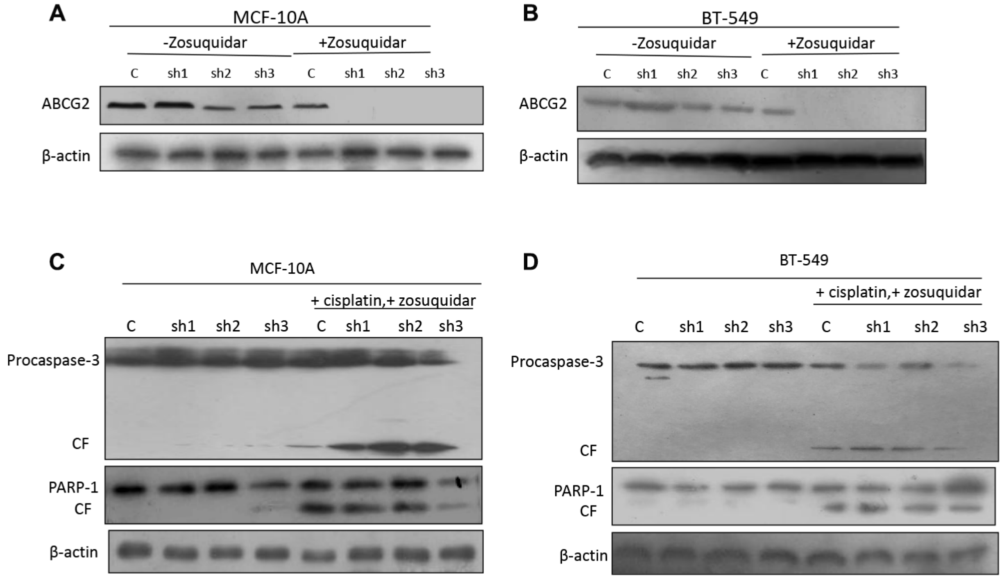

Supplementary Figure 6: Zosuquidar, a p-gp pump drug efflux inhibitor promoted the apoptotic effect of cisplatin in knockdown of AKT1 (shAKT1). (**A**, **B**) Western blot analysis of ABCG2 expression by p-gp inhibitor, zosuquidar. (**C**, **D**) Western blot images of apoptotic protein, Procaspase-3 and PARP-1 in AKT isoforms knockdown in (C) MCF-10A and (d) BT-549 respectively. Following 24 h pre-treatment with 2.5 µM zosuquidar, cells were exposed to cisplatin.

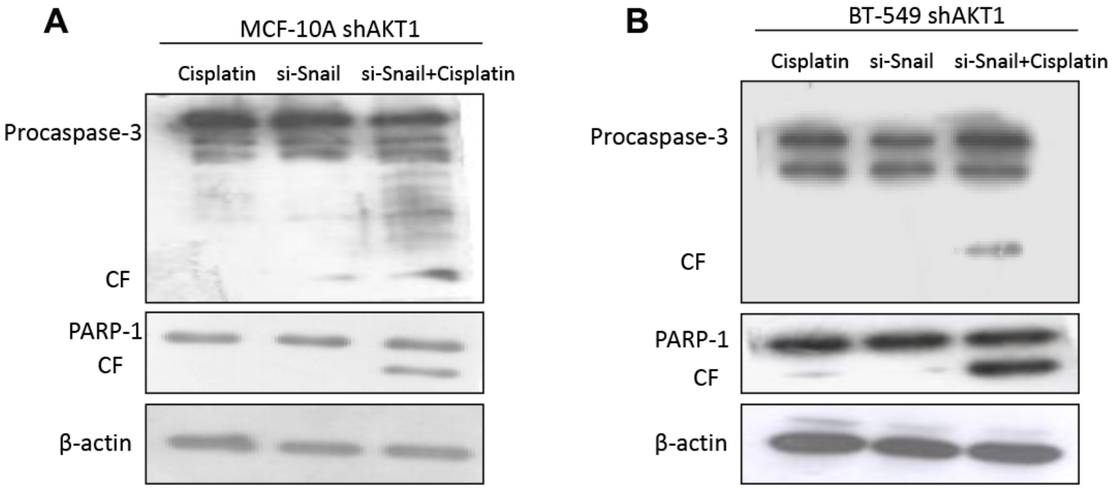

Supplementary Figure 7: Western blot images of the expression of anti-apoptosis. (**A**) Knockdown of Snail in shAKT1 MCF-10A and (**B**) BT-549 altered the expression of apoptotic-related proteins, Procaspase-3 and PARP-1.
